# Hepatitis B Virus Alters the Antioxidant System in Transgenic Mice and Sensitizes Hepatocytes to Fas Signaling

**DOI:** 10.1371/journal.pone.0036818

**Published:** 2012-05-11

**Authors:** Qian Wang, Bing Na, Jing-hsiung James Ou, Lynn Pulliam, T. S. Benedict Yen

**Affiliations:** 1 Department of Pathology, University of California San Francisco, San Francisco, California, United States of America; 2 Pathology Service 113B, VA Medical Center, San Francisco, California, United States of America; 3 Department of Molecular Microbiology and Immunology, University of Southern California, Los Angeles, California, United States of America; 4 Department of Laboratory Medicine, University of California San Francisco, VA Medical Center, San Francisco, California, United States of America; Institut Pasteur, France

## Abstract

Hepatitis B virus (HBV) is a major etiological factor of hepatocellular carcinoma (HCC). However, the precise pathogenetic mechanisms linking HBV infection and HCC remain uncertain. It has been reported that decreased antioxidant enzyme activities are associated with severe liver injury and hepatocarcinogenesis in mouse models. It is unclear if HBV can interfere with the activities of antioxidant enzymes. We established a HBV transgenic mouse line, which spontaneously developed HCC at 2 years of age. We studied the activities of the antioxidant enzymes in the liver of the HBV transgenic mice. Our results showed that the antioxidant enzymes glutathione peroxidase and superoxide dismutase 2 were down-regulated in HBV transgenic mice and correlated with JNK activation. HBV enhanced the Fas-mediated activation of caspase 6, caspase 8 and JNK without enhancing the activation of caspase 3 and hepatocellular apoptosis. As a proper redox balance is important for maintaining cellular homeostasis, these effects of HBV on the host antioxidant system and Fas-signaling may play an important role in HBV-induced hepatocarcinogenesis.

## Introduction

Intracellular reactive oxygen species (ROS) levels are tightly controlled by four primary antioxidant enzymes superoxide dismutase (SOD) 1 and 2 (CuZnSOD and MnSOD), glutathione peroxidase (GPx) and catalase. Superoxide radicals are converted into hydrogen peroxide by SOD 1 and 2, and hydrogen peroxide is converted into water by catalase and GPx [1], [2]. Oxidative stress can result from increased production of ROS and/or diminished levels of antioxidants including the activities of antioxidant enzymes [3].

Hepatocellula carcinoma (HCC) is the third leading cause of cancer death [4]. There are several published reports to show that decreased antioxidant enzyme activities are associated with severe liver injury and hepatocarcinogenesis in mouse models. For example, SOD1 deficient (*Sod1−/−*) mice have increased incidence to develop liver nodular hyperplasia or HCC [5]. GPx deficient (*Gpx−/−*) mice are more susceptible to liver injury-induced by tumor necrosis factor α (TNFα), anti-Fas antibody and oxidant paraquat [6], [7], [8]. Moreover, when challenged with oxidant paraquat, C-Jun amino-terminal kinases (JNK) are more activated in GPx deficient mice than in wild type mice [7]. The correlation between aberrant activation of JNK and hepatocarcinogenesis is observed in liver-specific deletion of p38α and IKKβ mouse models [9], [10]. In a liver-specific deletion of IKKβ mouse model, diethylnitrosamine (DEN)-induced ROS production may contribute to sustained JNK activation due to oxidation and inhibition of JNK-inactivating phosphatases [11]. JNK1 has also been reported to be activated in human HCC tissues compared to adjacent non-cancerous tissues, among which 70% are with hepatitis B virus (HBV) infection. However, the mechanism for JNK1 activation is unknown [12].

Both HBV and hepatitis C virus (HCV) are the major etiological factors of HCC [13], [14]. Lipid peroxidation has been detected in both HBV and HCV chronically infected patients, especially those with cirrhosis [15], [16]. Both *in vitro* and *in vivo* evidences demonstrate that HCV core protein can induce ROS production and corresponding elevated antioxidant response as shown by up-regulation of the mRNA of antioxidant genes, including GPx, increased catalase activity and decreased total and reduced glutathione level [17], [18]. However, it is unclear if HBV can interfere with the activities of antioxidant enzymes.

We established a HBV transgenic mouse line, which carries the 1.3 mer over-length HBV genome and productively replicates HBV in the liver. Due to immune tolerance, the HBV transgenic mice do not have liver inflammation until they spontaneously develop HCC at 2 years of age [19], [20]. We studied the activation of JNK and the activities of the antioxidant enzymes in the liver of the HBV transgenic mice. We found that both JNK1 and JNK2 were activated in the liver of the HBV transgenic mice. The activation of JNK was correlated with the down-regulation of the antioxidant enzymes SOD2 and GPx. The altered antioxidant enzyme system sensitized the HBV transgenic mice to the Fas signaling pathway.

## Results

### Activation of JNK in HBV transgenic mice

**Figure 1:**
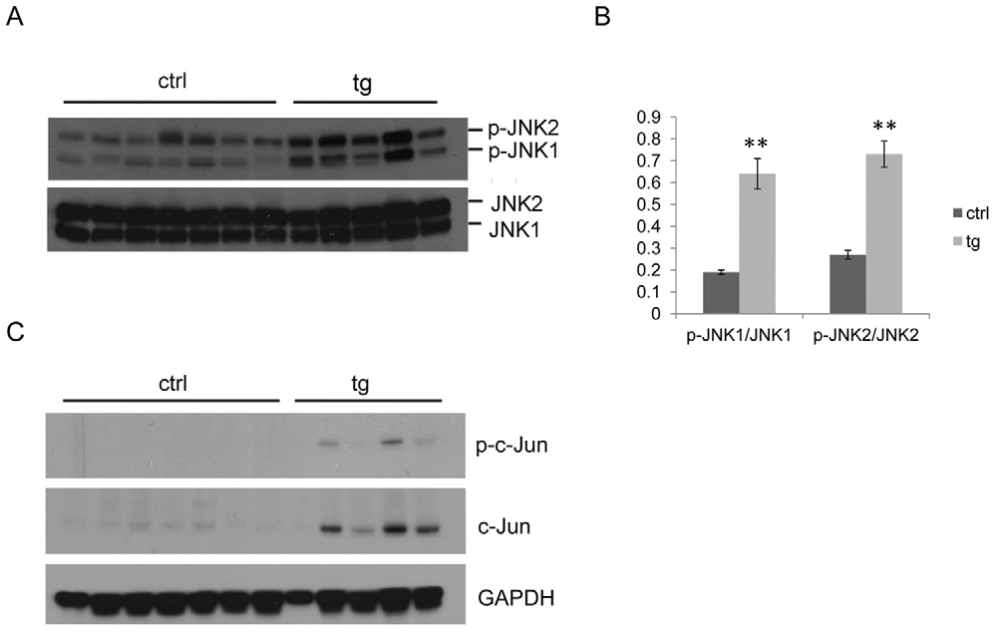
Activation of JNK in the liver of 2-year old HBV transgenic mice. (A) Western blot analysis of phosphorylated and total JNK in control (n = 7) and HBV transgenic (n = 5) mice. (B) Quantification of the activation of JNK1 and JNK2 (mean ± SEM) in control and HBV transgenic mice described in (A). Significant differences between control and HBV transgenic mice are indicated by two (*P*<0.01) asterisks. (C) Western blot analysis of phosphorylated and total c-Jun in the same control and HBV transgenic mice as described in (A).

The HBV transgenic mice spontaneously develop hepatocellular neoplasms, including HCC at two years of age [19]. Since activation of JNK has been observed in HCC mouse models and HCC patients [9], [10], [12], five HBV transgenic mice and seven non-transgenic control mice at two years of age were carefully identified by genotyping and chosen to study the activation of JNK in the liver. All of the five HBV transgenic mice developed HCC. Representative hematoxylin and eosin stained sections of HBV transgenic mouse liver with HCC and non-transgenic control mouse liver are shown in Fig. S1. First, we determined whether or not JNK is activated in the liver of 2-year-old HBV transgenic mice, which developed HCC, and non-transgenic control mice by analyzing the levels of JNK1, JNK2 and their activated forms (i.e., p-JNK1 and p-JNK2) by Western blot (Fig. 1A). There was no significant difference of total JNK1 and JNK2 levels between control mice and HBV transgenic mice. However, when the levels of p-JNK1 and p-JNK2 were quantified and normalized against their respective total JNKs, higher levels of p-JNK1 and p-JNK2 were detected in HBV transgenic mice (*P*<0.01) (Fig. 1B). Secondly, we analyzed the protein levels of phosphorylated c-Jun and total c-Jun in the same mice. c-Jun is one of the targets of activated JNK and demonstrated to be essential for development of liver tumors [23], [24]. Phosphorylated c-Jun can bind to its own promoter and enhance its own expression [25], [26]. As shown in Fig. 1C, among the five HBV transgenic mice, three of them showed increased phosphorylated c-Jun and four of them showed increased total c-Jun compared to control mice.

### Down-regulation of antioxidant enzymes GPx and SOD2 in HBV transgenic mice

**Figure 2:**
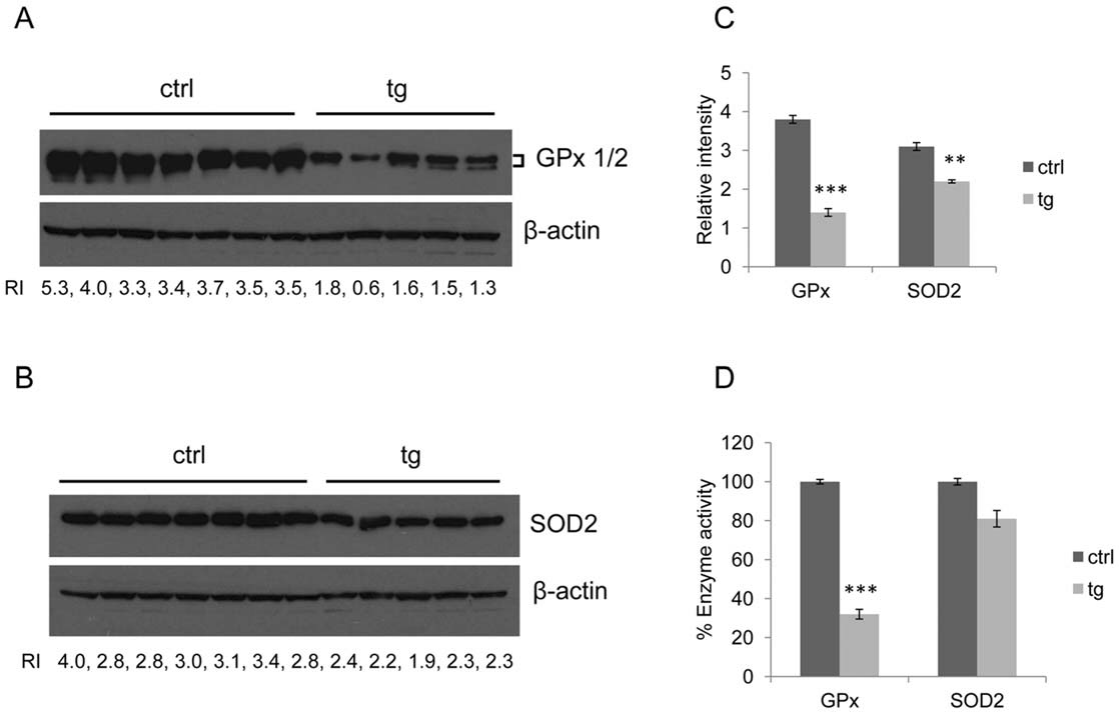
Down-regulation of antioxidant enzymes GPx and SOD2 in HBV transgenic mice. (A) Western blot analysis of GPx in the liver of the same control and HBV transgenic mice as described in Figure 1. Relative intensity (RI) refers to the intensity of GPx normalized to that of actin, which was used as the loading control. (B) Western blot analysis of SOD2 in the liver of the same control and HBV transgenic mice as described in Figure 1. RI refers to the intensity of SOD2 relative to that of actin. (C) Mean values of the RIs of GPx (mean ± SEM) and SOD2 (mean ± SEM) in control and HBV transgenic mice described in (A) and (B). Significant differences between control and HBV transgenic mice are indicated by three (*P*<0.0001) and two (*P*<0.01) asterisks. (D) Relative enzymatic activities of GPx and SOD2 in the same control and HBV transgenic mice as described in (A) and (B). The enzyme activities of GPx and SOD2 in the liver of control and HBV transgenic mice were measured and normalized to the protein concentrations. The enzyme activities of the control mice were arbitrarily defined as 100% (mean ± SEM). Significant differences between control and HBV transgenic mice are indicated by three asterisks (*P*<0.0001).

Since increased ROS production and decreased antioxidant defenses have been reported to be beneficial to activation of JNK [7], [10], [11], we examined antioxidant enzymes and lipid peroxidation in the liver of the same control and HBV transgenic mice as described above. First, we analyzed the protein levels of the antioxidant enzymes SOD1, SOD2, GPx and catalase. As shown in Fig. 2A and 2B, the protein levels of both GPx and SOD2 were lower in HBV transgenic mice than in control mice. The relative intensity (RI) of GPx or SOD2 to actin was calculated for individual mice and the mean was shown in Fig. 2C. The down-regulation of both GPx and SOD2 in HBV transgenic mice was statistically significant. Our results suggested that down-regulation of Gpx and SOD2 was correlated with activation of JNK in the liver of HBV transgenic mice.

To confirm these results, we also measured the enzyme activities of GPx and SOD2 in the mouse livers. The enzyme activities of GPx and SOD2 are shown in Table 1. The GPx and SOD2 activities were reduced by 70% and 20%, respectively, in HBV transgenic mice (Fig. 2D). The reduction of GPx activity, but not SOD2 activity in HBV transgenic mice was statistically significant. In contrast to GPx and SOD2, the expression levels of SOD1 and catalase were not significantly different between control mice and HBV transgenic mice (data not shown).

**Table 1:** Enzyme activities of GPx, SOD2 and MDA in the liver of control and HBV transgenic mice.

	GPx	SOD2	MDA
	(U/mg protein)	(U/mg protein)	(mM/mg protein)
**Control (n = 7)**	2.5±0.0	5.7±0.1	1.1±0.1
**tg (n = 5)**	0.8±0.1^***^	4.6±0.2	1.1±0.1

Values are mean ± SEM; significant difference between control and HBV transgenic mice are indicated by three asterisks (*P*<0.0001). tg = HBV transgenic mice.

**Figure 3:**
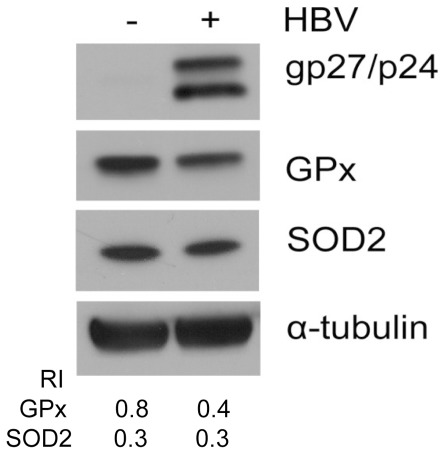
Suppression of GPx by HBV in C3A human hepatoma cells line. C3A cells transiently transfected with pHBV1.3 or empty vector for 48 h were lysed for Western blot analysis for HBV small surface proteins (glycosylated/unglycosylated, gp27/p24), GPx and SOD2. α-tubulin was also analyzed to serve as a loading control. The RIs of GPx and SOD2 to α-tubulin are shown in the lower panel.

To further investigate whether HBV has a direct effect on the expression levels of GPx and SOD2 in cell cultures, we transfected the HBV genome into the human hepatoma cell lines C3A and HuH-7. As shown in Fig. 3, the transfection of the HBV DNA genome reduced the expression of GPx but not SOD2 in C3A cells. These data indicated that HBV could directly suppress the expression of GPx but not SOD2 in C3A cells. We did not observe reduced expression of GPx and SOD2 in HuH-7 cells (data not shown).

We examined whether there was any difference in lipid peroxidation between control mice and HBV transgenic mice by measuring the levels of malondialdehyde (MDA) and found there was no difference in MDA levels between control mice and HBV transgenic mice (Table 1), indicating there was no lipid peroxidation in the liver of HBV transgenic mice.

### Enhancement of Fas-mediated activation of JNK in HBV transgenic mice

**Figure 4:**
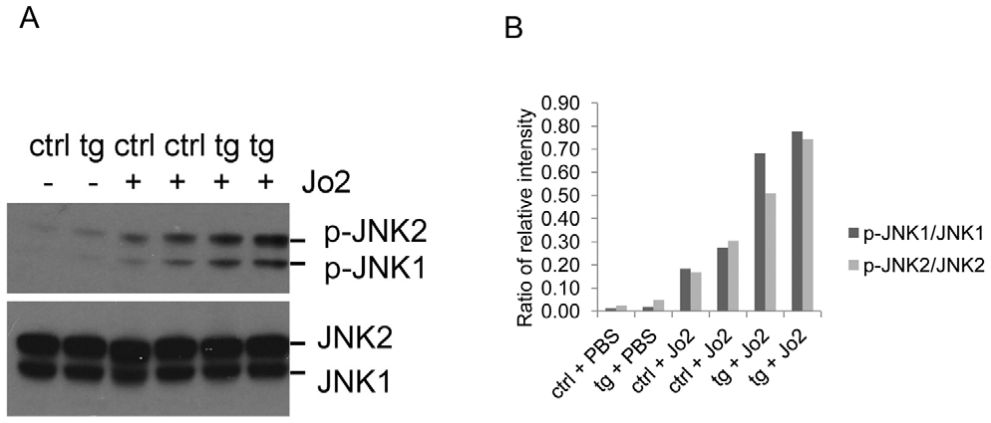
Fas-mediated activation of JNK in HBV transgenic mice. (A) Western blot analysis of activation of JNK in HBV transgenic mice and control mice. i.v. injected with PBS or anti-Fas antibody Jo2. (B) Quantification of activation of JNK1 and JNK2 in HBV transgenic mice and control mice as described in (A).

It has been reported that TNFα, anti-Fas antibody and oxidant paraquat can cause more severe liver injury in GPx-deficient (*Gpx−/−*) mice than in wild-type mice and JNK is more activated in paraquat-induced liver aponecrosis [6], [7], [8]. It has also been reported that anti-Fas antibody by intravenous injection will induce ROS production in the liver of the mouse [27]. We injected control and HBV transgenic mice intravenously with anti-Fas antibody Jo2, and investigated whether HBV transgenic mice were more susceptible to anti-Fas antibody-induced hepatocellular apoptosis. We also investigated the protein levels and enzyme activities of GPx, SOD2 and activation of JNK in this process. We analyzed the hepatocellular apoptosis by performing the TUNEL assay. The injection of the anti-Fas antibody Jo2 induced similar degrees of hepatocellular apoptosis in control mice and HBV transgenic mice (Fig. S2). The enzyme activities of GPx and SOD2 were lower in HBV transgenic mice than in control mice after anti-Fas antibody injection (Table 2). The protein levels of GPx and SOD2 are shown in Fig. S3. Aconitase can be inactivated by ROS and therefore its activity has been used as an indirect indicator of ROS [28]. We measured the aconitase activities of control and HBV transgenic mice injected with Jo2. The aconitase activity of HBV transgenic mice was 10% lower than that of control mice (Fig. S4), indicating that a compromised antioxidant system facilitates ROS production. Activation of JNK was observed in both control and HBV transgenic mice after anti-Fas antibody injection compared to the mice injected with saline, and the activation of JNK was more pronounced in HBV transgenic mice than in control mice when the mice were injected with the anti-Fas antibody (Fig. 4A). Quantification of activation of JNK is shown in Fig. 4B.

**Table 2:** Enzyme activities of GPx and SOD2 in the liver of control and HBV transgenic mice intravenously injected with anti-Fas antibody Jo2.

	GPx	SOD2
	(U/mg protein)	(U/mg protein)
**Control-1**	2.1±0.1	5.4±0.5
**Control-2**	2.1±0.0	6.7±0.1
**tg-1**	1.2±0.3	4.5±0.0
**tg-2**	0.8±0.5	3.7±0.1

Values are mean ± SD. tg = HBV transgenic mice.

### Enhancement of Fas-mediated activation of caspases 8 and 6 in HBV transgenic mice

**Figure 5:**
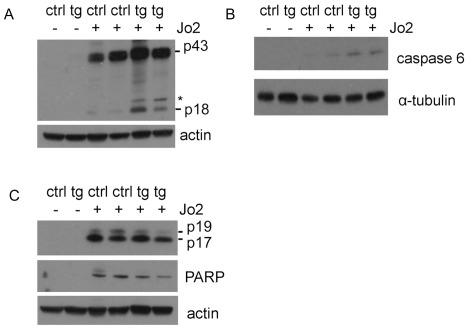
Fas-mediated activation of caspases 8, 6 and 3 in HBV transgenic mice. (A) Western blot analysis of cleaved caspase 8. The cleaved 43 kDa and 18 kDa caspase 8 protein bands are highlighted, *non specific protein band. RI refers to the intensity of cleaved p43 and p18 relative to that of actin. (B) Western blot analysis of cleaved caspase 6. RI refers to the intensity of caspase 6 relative to that of α-tubulin. (C) Western blot analysis of cleaved caspase 3 and PARP. The cleaved 19 kDa and 17 kDa caspase 3 protein bands are highlighted. RI refers to the intensity of cleaved p19, p17 and PARP relative to that of actin.

Although we did not observe the difference of hepatocellular apoptosis using a TUNEL assay between control and HBV transgenic mice, we analyzed activation of caspases, since activation of caspases occurs earlier than DNA fragmentation during apoptosis. Caspase 8 is the initiator caspase of Fas-triggered apoptosis and its autoproteolysis is essential for Fas-induced apoptosis [29], [30]. As shown in Fig. 5A, the cleaved caspase 8 (43 kD and 18 kD) was detected in HBV transgenic and control mice after being intravenously injected with anti-Fas antibody Jo2, but not in mice injected with saline. The cleaved p18 of caspase 8 was more pronounced in HBV transgenic mice than in control mice, indicating that HBV could sensitize hepatocytes to Fas signaling. To confirm this result, we analyzed the cleavage of caspase 6, which can also be triggered by the activation of Fas [31]. As shown in Fig. 5B, there was an increased level of cleaved caspase 6 in HBV transgenic mice than in control mice after the anti-Fas antibody injection. Caspase 6 is thought to be an effector caspase because it can cleave the nuclear structural protein NuMA and the lamin proteins. However, it has also been shown that caspase 6 cleaves itself, caspase 3 and 8 [32]. This result was consistent with the activation of caspase 8 and further indicated that HBV could sensitize hepatocytes to Fas signaling. Interestingly, caspase 3, the downstream effector of caspase 8 and 6, was activated to a lesser degree in HBV transgenic mice than in control mice, as revealed by the similar levels of the cleaved p17 of caspase 3 and diminished levels of cleaved p19 and the cleaved poly ADP-ribose polymerase (PARP), which is the downstream target of caspase 3 (Fig. 5C). As HBV could enhance the activation of caspases 8 and 6 and not enhance the activation of caspase 3 or Fas-mediated hepatocellular apoptosis, HBV apparently could enhance only the early apoptotic pathways in response to Jo2.

## Discussion

Our recent studies indicate that HBV could contribute directly to the development of HCC [19]. To date, the precise pathogenetic mechanisms linking HBV infection and HCC remain uncertain [14], [33]. With our HBV transgenic mouse model, we observed JNK activation in HBV transgenic mice, which developed HCC. This result is consistent with the result observed in human HCC, among which 70% were HBV positive [12]. Concomitant with JNK activation, antioxidant enzymes GPx and SOD2 were down-regulated in HBV transgenic mice. Down-regulation of GPx and SOD2 may compromise the ability to cope with ROS production in the liver of HBV transgenic mice. TNFα-induced ROS accumulation leads to sustained JNK activation due to lack of SOD2 in NFκB-deficient cells and oxidant paraquat can promote JNK activation in the liver of GPx-deficient mice [7], [11]. ROS function as signaling molecules in platelet-derived growth factor (PDGF) signaling [34], epidermal growth factor (EGF) signaling [35], insuling signaling [36] and vascular endothelial growth factor (VEGF) signaling [37]. However, excessive ROS is toxic and proper ROS level is important for maintaining cellular homeostasis [1], [38], [39], [40]. As HBV transgenic mice were intravenously injected with anti-Fas antibody, which can induce ROS production in the liver of the mouse [27], we observed that JNK was activated more profoundly in the liver of HBV transgenic mice and correlated with the lower activities of GPx and SOD2.

We observed JNK activation and down-regulation of GPx and SOD2 only in 2-year old HBV transgenic mice, but not in 9-month and younger mice (data not shown). Increasing age has been recognized as a correlate of decrease in response to liver injury and poor outcome in chronic liver disorders [41]. Decreased antioxidant defenses including the protein levels of antioxidant enzymes are associated with aging, and thus sensitize the cells, organs and organisms to oxidative damage [16], [42], [43], [44]. The down-regulation of GPx and SOD2 in HBV mice with old age indicated that the combination of HBV infection and aging may lead to accelerated damage of antioxidant system and hepatocarcinogenesis.

Caspase 8 is required for Fas-induced JNK activation [45], [46]. Caspase 6 can cleave caspase 8 [32]. These enzymes play important roles in the promotion of apoptosis. The injection of the anti-Fas antibody Jo2 generated a stronger activation response of caspase 8, caspase 6 and JNK in HBV transgenic mice than in control mice. Interestingly, in spite of the activation of these enzymes, there was no increased activation of caspase 3 or apoptosis in the liver of HBV transgenic mice. These results indicated that HBV could sensitize hepatocytes to Fas ligands to elicit a partial apoptotic response but it was not able to sensitize hepatocytes to apoptosis induced by Fas. Our results are reminiscent of previous reports, which indicated that the anti-Fas antibody could induce higher plasma alanine transaminase (ALT) values in *Gpx*−/− mice than in wild-type mice without increasing the caspase 3 activities [6]. Since GPx was down-regulated by HBV, it is conceivable that this reduction of GPx activity by HBV may be partially responsible for the lack of enhancement of caspase 3 activation. It has also been reported that HBx protein can protect cells from Fas-induced apoptosis and this protection requires activation of JNK [47], [48]. HBx protein may be another reason for the lack of enhanced caspase 3 activation.

In conclusion, our results demonstrated that HBV could affect the antioxidant enzymes in the mouse liver and sensitize hepatocytes to Fas signaling. This alteration of the antioxidant system and the sensitization to Fas signaling may contribute to hepatocarcinogenesis induced by HBV.

## Materials and Methods

### Mice

HBV transgenic mice used in this study have been described previously [20]. These mice were backcrossed to C57BL/6J for at least 6 generations. The mice were housed in the animal facility at the Veterans Affairs Medical Center, San Francisco (VAMCSF). All procedures were approved by the Institutional Animal Care and Use Committee (IACUC) at the VAMCSF and carried out in accordance with the IACUC policies. To study the effect of the anti-Fas antibody, mice were intravenously (i.v.) injected with 120 µl phosphate buffered saline (PBS) with or without Jo2 antibody (0.6 mg/kg, BD Pharmingen). Two and a half hours after injection, mice were euthanized with carbon dioxide (CO_2_), followed by cervical dislocation. The liver tissues were removed, rinsed with PBS, and either fixed in 10% buffered formalin (Fisher) for TUNEL (terminal deoxynucleotidyl transferase dUTP nick end labeling) assay and histological analyses or flash frozen in liquid nitrogen and then stored at −80°C for Western blot and enzyme assays. Apoptosis was detected by the TUNEL assay with the Apop Tag Peroxidase In Situ Apoptosis Detection Kit (Millipore, Cat. S7100).

### Western blot analyses

Frozen liver tissues (20 µg) were washed in ice cold PBS, and then homogenized at 4°C in 80 µl 50 mM Tris-HCl (pH 8.0) containing the protease inhibitor cocktail (Fermentas) and the phosphatase inhibitor cocktail (Roche) with the Bullet Blender machine (Next Advance Inc.). The tissue homogenates were freeze-thawed 3 times to disrupt the mitochondrial membrane [5]. Protein concentrations of tissue homogenates were determined with the Bradford assay (Bio-Rad). 5 to 40 µg protein was loaded on a 4–20% SDS-PAGE gradient gel (TGX gel, Bio-Rad). Anti-GPx (B-6) and anti-catalase (11A1) antibodies were purchased from Santa Cruz Biotechnology; anti-SOD1 (Cat. AB5482) and anti-SOD2 (Cat. 06-984) antibodies were purchased from Millipore; anti-actin (Ab-1) mouse mAb (JLA20) was purchased from Calbiochem. The apotosis antibody sampler kit (mouse specific), the phospho- MAPK family antibody sampler kit, the MAPK family antibody sampler kit, the anti-cleaved caspase-8 (Asp387) antibody (mouse specific), the anti-c-Jun antibody (60A8), the anti-GAPDH (14C10) antibody, and the anti-α-tubulin (11H10) antibody were all purchased from Cell Signaling Technology. The goat anti-HBV surface antigen (Ad/Ay) antibody was purchased from Abcam. All secondary antibodies were purchased from Santa Cruz Biotechnology. Western blot results were quantified using Image J (NIH) software.

### Enzyme assays and lipid peroxidation assay

For all enzyme assays, the frozen liver tissues were washed with ice cold PBS, and homogenized at 4°C in the buffers provided by the individual enzyme assay kits. The homogenates were freeze-thawed 3 times using dry ice and room temperature water bath. Protein concentrations were determined by the Bradford assay and adjusted to 0.5 mg/ml. GPx activities were determined with the Glutathione Peroxidase Cellular Activity Assay Kit (Sigma-Aldrich, Cat. CGP1). SOD activities were determined with the SOD Determination Kit (Sigma-Aldrich, Cat. 19160). The GPx standard (glutathione peroxidase from bovine erythrocytes) and SOD standard (superoxide dismutase from human erythrocytes, Cat. S9636) were purchased from Sigma-Aldrich. To determine the SOD2 activities, the homogenates were incubated with potassium cyanide (final concentration 5 mM) for 30 min at room temperature to inactivate SOD1 [2], [21]. Aconitase activities were determined with Aconitase Assay Kit (Cayman Chemical Com., Cat. 705502). The lipid peroxidation levels were determined with OxiSelect TBARS Assay Kit (MDA Quantification) (Cell Biolabs Inc., Cat. STA-330). All the readings were performed with the Modulus Microplate Multimode Plate Reader (Turner BioSystems). All the enzymatic activities measured were normalized to the protein concentrations.

### Plasmids, cell culture and transfection

The pHBV1.3 plasmid contains the 1.3mer over-length HBVgenome in the pUC19 vector. This plasmid contains the entire HBV genome plus a terminal redundancy of nearly 1 kb [22]. Human hepatoma cell line C3A was purchased from American Type Culture Collection (ATCC) and cultured in Eagle's Minimum Essential (MEM) medium supplemented with non-essential amino acids, 2 mM L-glutamine, 1 mM sodium pyruvate, 1.5 g/L sodium bicarbonate and 10% fetal bovine serum at 37°C in 5% CO_2_. C3A cells (1.5×10^5^) were seeded into 24-well plates. The cells were transfected with 400 ng pUC19 or pHBV1.3 using the FuGENE HD transfection reagent (Roche). Forty-eight hours post transfection, cells were trypsinized, washed with PBS and lysed in the RIPA buffer (1% Nonidet P-40, 0.25% sodium deoxycholate, 150 mM NaCl, and 50 mM Tris-HCl pH 7.4) containing the protease inhibitor cocktail (Fermentas) and the phosphatase inhibitor cocktail (Roche). After a brief centrifugation to remove cell debris, the supernatants were collected for Western blot analysis.

### Statistical analysis

Student's t- test was used to analyze the data. *P* values were calculated in individual assays and *P*<0.05 was considered as statistically significant.

## Supporting Information

Figure S1 **The representative hematoxylin and eosin stained sections.** (A) Non-tg control mouse liver section. (B) HBV tg mouse liver section with HCC.
(TIF)

Figure S2 **Mouse liver apoptosis detected by TUNEL assay.** (A) Non-tg control mouse was i.v. injected with 120 µl PBS. (B) HBV transgenic mouse was i.v. injected with 120 µl PBS. (C–D) Two non-tg control mice were i.v. injected with anti-Fas antibody Jo2 (0.6 mg/kg) in 120 µl PBS. (E–F) Two HBV tg mice were i.v. injected with anti-Fas antibody Jo2 (0.6 mg/kg) in 120 µl PBS. The mice were sacrificed 2.5 h post injection. Paraffin-embedded liver samples were stained for apoptotic cells with TUNEL assay. (G) The apoptotic cells in ten individual fields of two non-tg control mice and two HBV tg mice were counted and normalized to the surface area (mean ± SD).
(TIF)

Figure S3 **Protein levels of GPx and SOD2 in the liver of non-transgenic control and HBV transgenic mice injected with Jo2.** (A) Western blot analysis of GPx in the liver of control and HBV transgenic mice. RI refers to the intensity of GPx normalized to that of α-tubulin, which was used as the loading control. (B) Western blot analysis of SOD2 in the liver of control and HBV transgenic mice. RI refers to the intensity of SOD2 relative to that of α-tubulin.
(TIF)

Figure S4 **Analysis of aconitase activities in the liver of control and HBV transgenic mice injected with Jo2.** Aconitase activities were measured in two control and two HBV tg mice and normalized to the protein concentrations. The aconitase activity of control mice were arbitrarily defined as 100% (mean ± SD).
(TIF)
