# Supplementary figures and images for: Hepatitis B Virus Alters the Antioxidant System in Transgenic Mice and Sensitizes Hepatocytes to Fas Signaling
Source: PLoS One. 2012 May 11;7(5):e36818. doi: 10.1371/journal.pone.0036818 (PMC3350475; doi:10.1371/journal.pone.0036818)

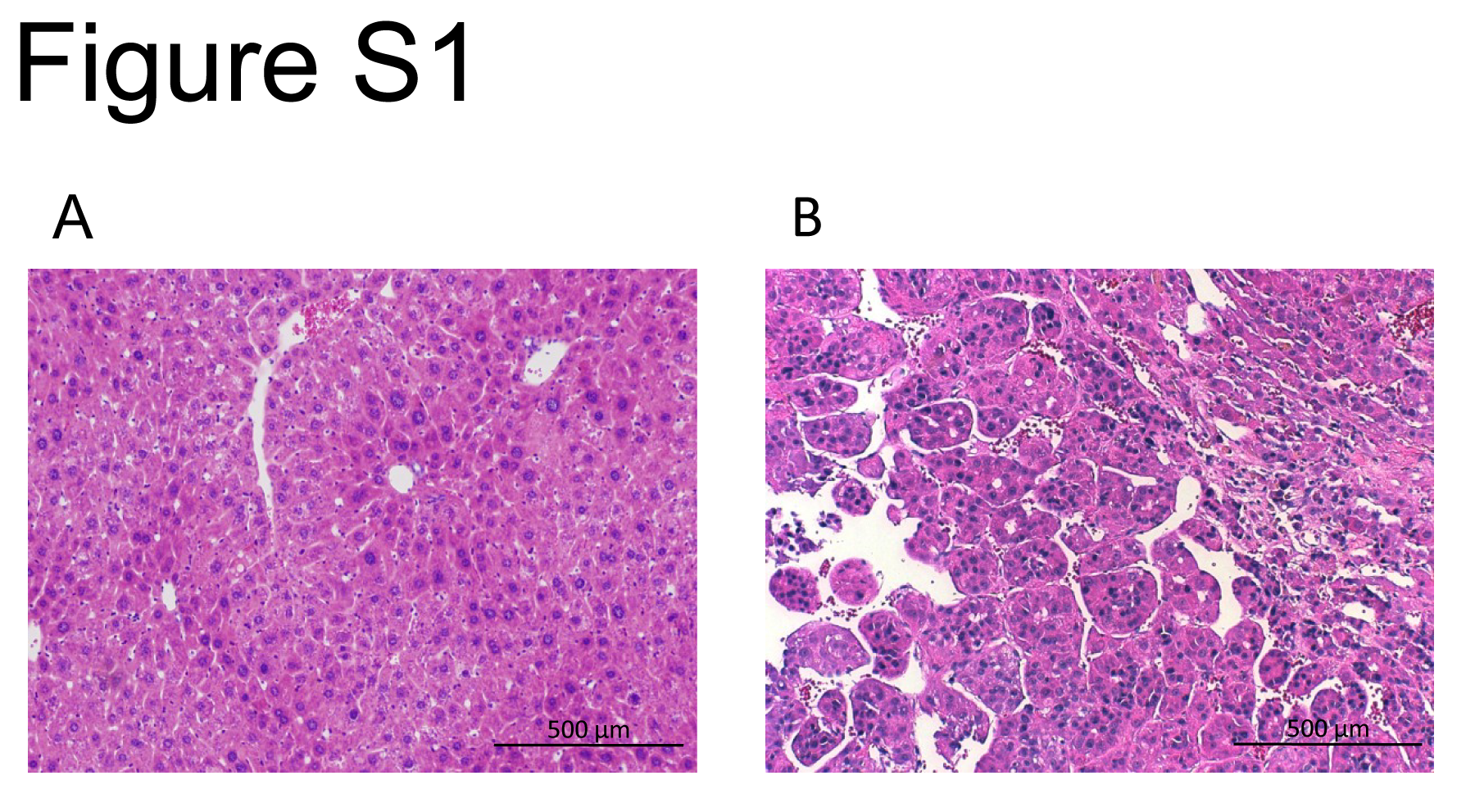

Supplement: Figure S1 — The representative hematoxylin and eosin stained sections. (A) Non-tg control mouse liver section. (B) HBV tg mouse liver section with HCC. (TIF) [file pone.0036818.s001.tif]

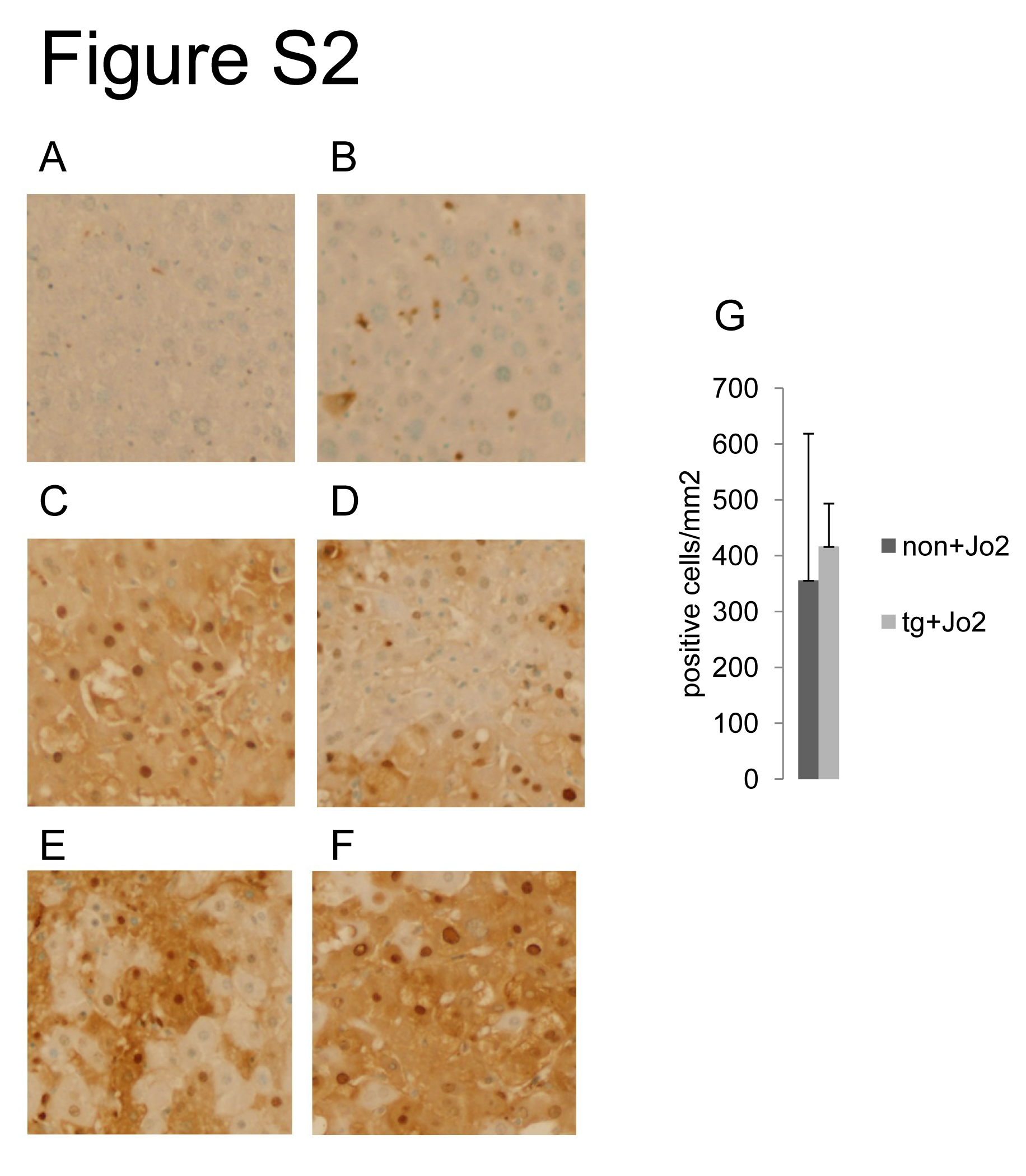

Supplement: Figure S2 — Mouse liver apoptosis detected by TUNEL assay. (A) Non-tg control mouse was i.v. injected with 120 µl PBS. (B) HBV transgenic mouse was i.v. injected with 120 µl PBS. (C–D) Two non-tg control mice were i.v. injected with anti-Fas antibody Jo2 (0.6 mg/kg) in 120 µl PBS. (E–F) Two HBV tg mice were i.v. injected with anti-Fas antibody Jo2 (0.6 mg/kg) in 120 µl PBS. The mice were sacrificed 2.5 h post injection. Paraffin-embedded liver samples were stained for apoptotic cells with TUNEL assay. (G) The apoptotic cells in ten individual fields of two non-tg control mice and two HBV tg mice were counted and normalized to the surface area (mean ± SD). (TIF) [file pone.0036818.s002.tif]

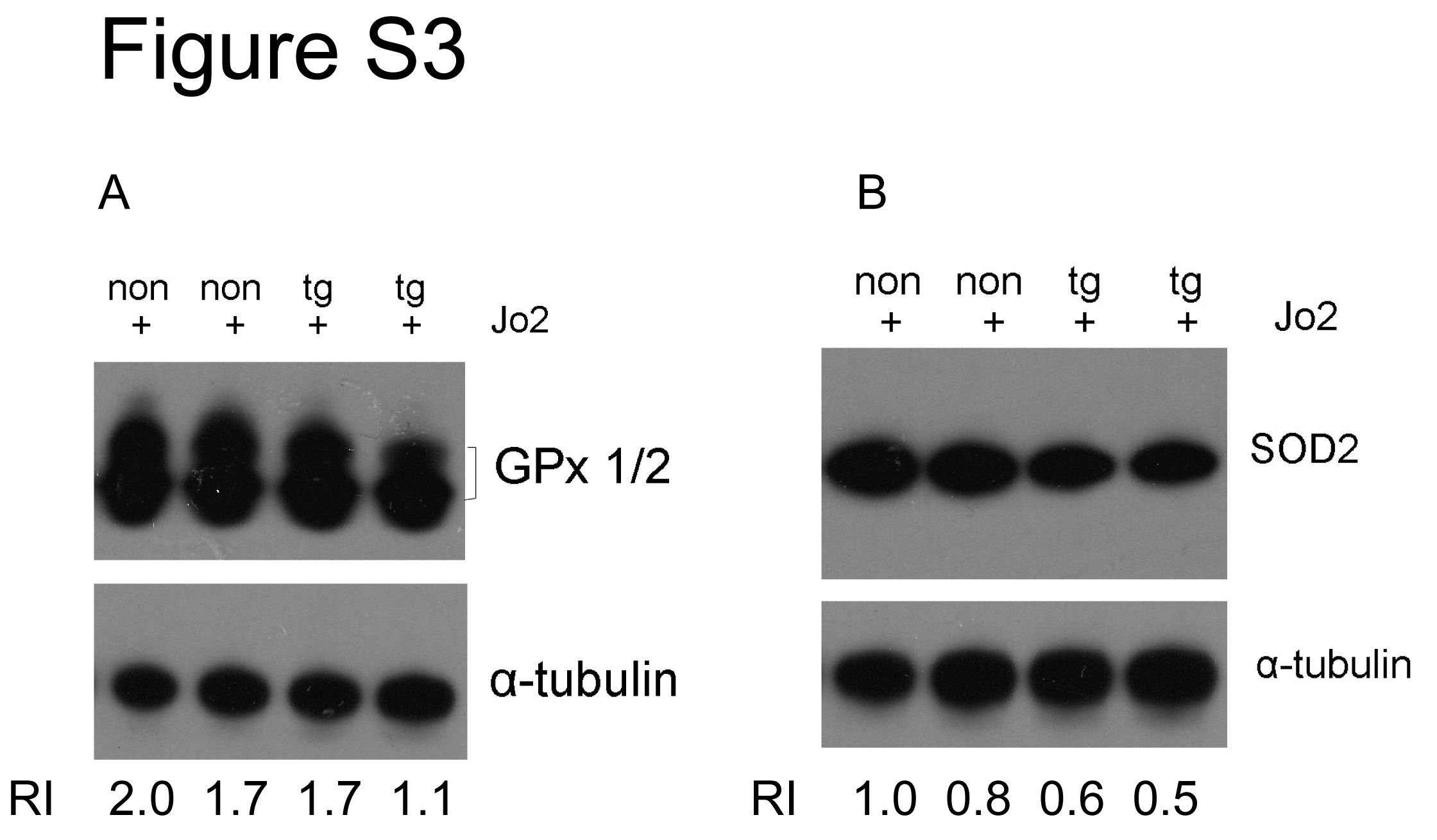

Supplement: Figure S3 — Protein levels of GPx and SOD2 in the liver of non-transgenic control and HBV transgenic mice injected with Jo2. (A) Western blot analysis of GPx in the liver of control and HBV transgenic mice. RI refers to the intensity of GPx normalized to that of α-tubulin, which was used as the loading control. (B) Western blot analysis of SOD2 in the liver of control and HBV transgenic mice. RI refers to the intensity of SOD2 relative to that of α-tubulin. (TIF) [file pone.0036818.s003.tif]

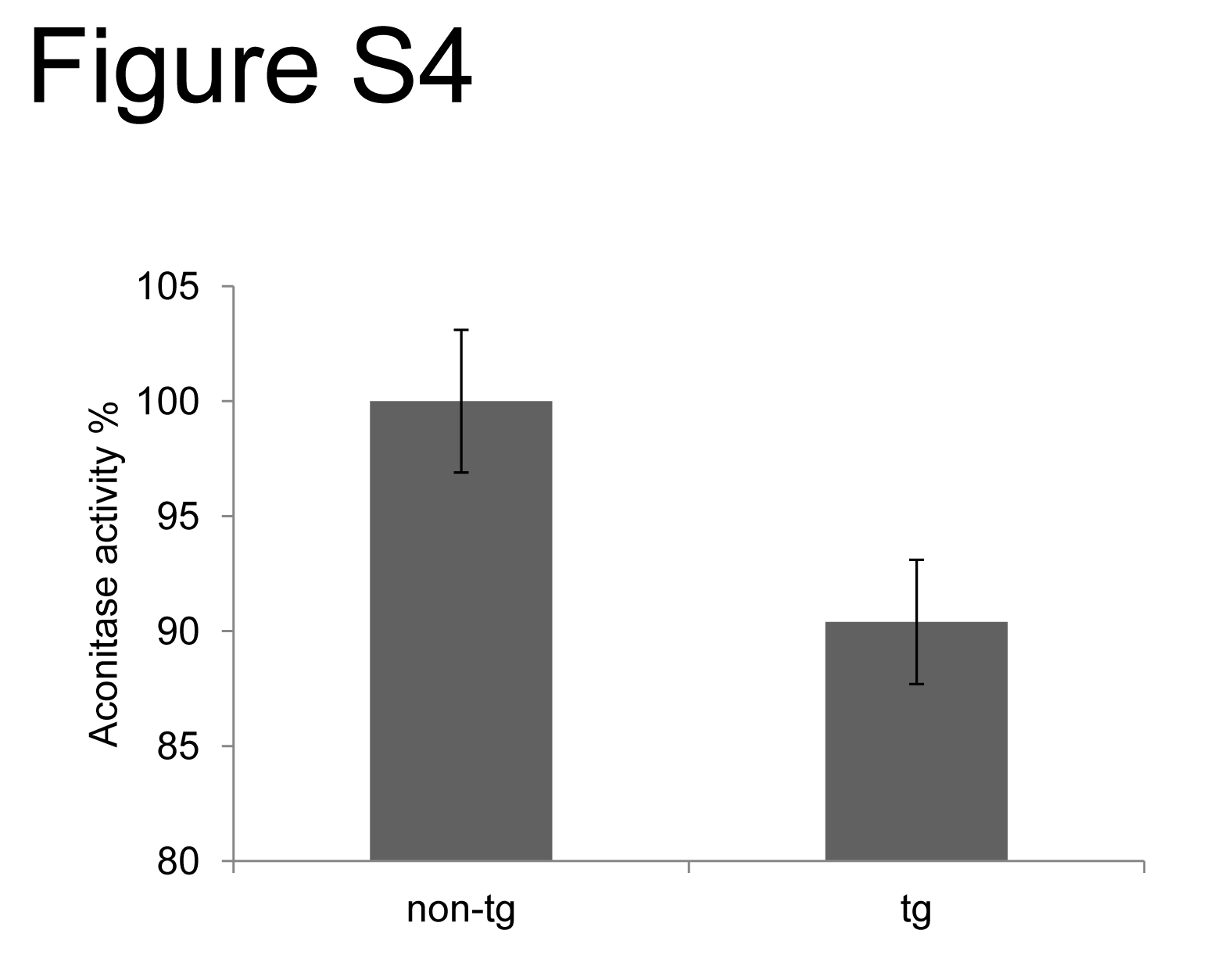

Supplement: Figure S4 — Analysis of aconitase activities in the liver of control and HBV transgenic mice injected with Jo2. Aconitase activities were measured in two control and two HBV tg mice and normalized to the protein concentrations. The aconitase activity of control mice were arbitrarily defined as 100% (mean ± SD). (TIF) [file pone.0036818.s004.tif]
